# Supplementary material for: Evaluating the Relationship between Well-Being and Living with a Dog for People with Chronic Low Back Pain: A Feasibility Study
Source: Int J Environ Res Public Health. 2019 Apr 25;16(8):1472. doi: 10.3390/ijerph16081472 (PMC6517939; doi:10.3390/ijerph16081472)
Supplement: Supplementary file 1 [file ijerph-16-01472-s001.zip › ijerph-468433-Supplementary Materials/Supplementary file_FINAL Codebook Nov 1 2017.docx]

# **CODEBOOK for**

# **Questionnaire Measures for Evaluating the Benefits of**

# **Dog Ownership for People with Chronic Low Back Pain**

**october 7 2017**

**SURVEY id Number Coding 000-999 ID**

**SECTION ONE: WELL BEING/QUALITY OF LIFE**

***Compared to other people your age, how would you rate your…***

overall health? **OHEALTH**

physical health? **PHEALTH**

mental or emotional health? **MHEALTH**

**Coding:** poor =1; fair = 2; good = 3; very good = 4; excellent = 5 (missing=9)

***Healthy Days Measure (Centers for Disease Control and Prevention, 2000)***

Please fill out how many days in the past 30 days…

- was your physical health poor? **HDPHYS**

**Coding: code actual numbers from 00-30 (99=missing)**

- was your mental health poor? **HDMENT**

**Coding: code actual numbers from 00-30 (99=missing)**

- did your physical or mental health keep you from doing your usual activities, such as self-care, work, or recreation? **HDPREV**

**Coding: code actual numbers from 00-30 (99=missing)**

**Recoding: UNHDAY (Unhealthy Days) = HDPHYS + HDMENT to a maximum of 30**

**Recoding: HDAYTOT (Healthy Days) = 30-UNHDAY**

***Pain Severity: Numeric Pain Intensity Scale (Salaffi et al., 2004, 2015)* PAINSEV**

Please indicate which of the numbers from 0 to 10 best describes your pain now (or over the last week).

**Worst**

**No** | _____|_____|_____|_____|_____|_____|_____|_____|_____|_____| **Possible**

**Pain** | | | | | | | | | | | **Pain**

0 1 2 3 4 5 6 7 8 9 10

**Coding: code actual numbers from 00-10 (99=missing)**

***Physical Functioning: Modified Oswestry Low Back Pain Questionnaire (Roland and Fairbank, 2001)***

This questionnaire is designed to enable us to understand how much you low back pain has affected your ability to manage your everyday activities. Please answer each section by marking in each section **one circle** that most applies to you. We realize that you may feel that more than one statement may relate to you, but please **just mark the circle that most closely describes your problem.**

## Section 1 - Pain Intensity PFPAIN

**O** The pain comes and goes and is very mild. = 0

**O** The pain is mild and does not vary much. = 1

**O** The pain comes and goes and is moderate. = 2

**O** The pain is moderate and does not vary much. = 3

**O** The pain comes and goes and is severe. = 4

**O** The pain is severe and does not vary much. = 5

(9 = missing)

## Section 2 - Personal Care PFCARE

**O** I do not have to change my way of washing or dressing to avoid pain. = 0

**O** I do not normally change my way of washing or dressing even though it causes me pain. = 1

**O** Washing and dressing increase the pain, but I manage not to change my way of doing it. = 2

**O** Washing and dressing increases the pain and I find it necessary to change my way of doing it. = 3

**O** Because of the pain I am unable to do some washing and dressing without help. = 4

**O** Because of the pain I am unable to do any washing and dressing without help. = 5

(9 = missing)

## Section 3 - Lifting (skip if you have not attempted lifting since the onset of your low back pain) PFLIFT

**O** I can lift heavy weights without extra low back pain. = 0

**O** I can lift heavy weights but it causes extra pain. = 1

**O** Pain prevents me lifting heavy weights off the floor. = 2

**O** Pain prevents me lifting heavy weights off the floor, but I can manage if they are conveniently positioned, e.g. on a table. = 3

**O** Pain prevents me lifting heavy weights but I can manage light to medium weights if they are conveniently positioned. = 4

**O** I can only lift light weights at the most. = 5

(9 = missing)

## Section 4 – Walking PFWALK

**O** I have no pain walking. = 0

**O** I have some pain on walking, but I can still walk my required to normal distances. = 1

**O** Pain prevents me from walking long distances. = 2

**O** Pain prevents me from walking intermediate distances. = 3

**O** Pain prevents me from walking even short distances. = 4

**O** Pain prevents me from walking at all. = 5

(9 = missing)

## Section 5 – Sitting PFSIT

**O** Sitting does not cause me any pain. = 0

**O** I can sit as long as I need provided I have my choice of sitting surfaces. = 1

**O** Pain prevents me from sitting more than 1 hour. = 2

**O** Pain prevents me from sitting more than 1/2 hour. = 3

**O** Pain prevents me from sitting more than 10 minutes. = 4

**O** Pain prevents me from sitting at all. = 5

(9 = missing)

## Section 6 – Standing PFSTAND

**O** I can stand as long as I want without pain. = 0

**O** I have some pain while standing, but it does not increase with time. = 1

**O** I cannot stand for longer than 1 hour without increasing pain. = 2

**O** I cannot stand for longer than 1/2 hour without increasing pain. = 3

**O** I cannot stand for longer than 10 minutes without increasing pain. = 4

**O** I avoid standing because it increases the pain immediately. = 5

(9 = missing)

## Section 7 – Sleeping PFSLEEP

**O** I have no pain while in bed. = 0

**O** I have pain in bed, but it does not prevent me from sleeping well. = 1

**O** Because of pain I sleep only 3/4 of normal time. = 2

**O** Because of pain I sleep only 1/2 of normal time. = 3

**O** Because of pain I sleep only 1/4 of normal time. = 4

**O** Pain prevents me from sleeping at all. =5

(9 = missing)

## Section 8 - Social Life PFSOCIAL

**O** My social life is normal and gives me no pain. = 0

**O** My social life in normal, but increases the degree of pain. = 1

**O** Pain prevents me from participating in more energetic activities e.g. sports, dancing. = 2

**O** Pain prevents me from going out very often. = 3

**O** Pain has restricted my social life to my home. = 4

**O** I hardly have any social life because of pain. =5

(9 = missing)

## Section 9 – Traveling PFTRAVEL

**O** I get no pain while traveling. = 0

**O** I get some pain while traveling, but none of my usual forms of travel make it any worse. = 1

**O** I get some pain while traveling, but it does not compel me to seek alternative forms of travel. = 2

**O** I get extra pain while traveling that requires me to seek alternative forms of travel. = 3

**O** Pain restricts all forms of travel. = 4

**O** Pain prevents all forms of travel except that done lying down. = 5

(9 = missing)

## Section 10 - Employment/Homemaking PFWORK

**O** My normal job/homemaking duties do not cause pain. = 0

**O** My normal job/homemaking duties cause me extra pain, but I can still perform all that is required of me. = 1

**O** I can perform most of my job/homemaking duties, but pain prevents me from performing more physically stressful activities e.g. lifting, vacuuming, etc. = 2

**O** Pain prevents me from doing anything but light duties. = 3

**O** Pain prevents me from doing even light duties. = 4

**O** Pain prevents me from performing any job or homemaking chore. = 5

(9 = missing)

**PFTOT *Scoring****: Questions are scored on a vertical scale of 0-5. Total scores and multiply by 2. Divide by number of sections answered multiplied by 10. A score of 22% or more is considered significant activities of daily living disability. (Score___ x 2) / (____Sections x 10) = %ADL*

**SECTION TWO: PHYSICAL ACTIVITY AND PHYSICAL HEALTH**

***Leisure Time in Exercise (Brown and Rhodes, 2006 adaptation Godin and Shepard, 1985)******.***

1) Considering a typical **7-day period** (a week), how many times on average do you do the following kinds of exercise for **more than 15 minutes** during your **free time**? Please write the appropriate number in the spaces below.

**Strenuous Exercise (Heart Beats Rapidly**) (i.e., running, jogging, hockey, football, soccer, squash, basketball, cross country skiing, judo, roller skating, vigorous swimming, vigorous long distance cycling).

_____ times per week **STRENXT** for _____ minutes each time **STRENXM**

**Coding**: 000 to 998 (missing=999) times per week for 000 to 998 minutes (missing=999)

**Moderate Exercise (Not Exhausting)** (i.e., fast walking, baseball, tennis, easy cycling, volleyball, badminton, easy swimming, alpine skiing, popular and folk dancing)

_____ times per week **MODXT** _____ minutes each time **MODXM**

**Coding**: 000 to 998 (missing=999) times per week for 000 to 998 minutes (missing=999)

**Mild Exercise (Minimal Effort)** (i.e., yoga, archery, fishing from river bank, bowling, horseshoes, golf, snow mobiling, easy walking)

_____ times per week **MINXT** _____ minutes each time **MINXM**

**Coding**: 000 to 998 (missing=999) times per week for 000 to 998 minutes (missing=999)

**Recoding: TOTALEXT** Total Weekly Leisure Activity calculation = (9 x strenuous times) + (5 x moderate times) + (3 x light times)

2) Considering a typical **7-day period** (a week), during your leisure time, how often do you engage in any regular activity long enough to **work up a sweat** (heart beats rapidly)? **SWEAT**

**Coding**: Never (1), not very often (2), sometimes (3), often (4), most of the time (5) (missing=9)

***Leisure Time Walking (******Brown and Rhodes, 2006 adaptation of Godin and Shepard, 1985)***

Considering a typical **7-day period** (a week), how many times on average do you walk for **more than 15 minutes** during your **free time with or without a dog**? Please write the appropriate numbers in the spaces below. If you do not own a dog, please write NA in the appropriate spaces below.

**Strenuous Walking (Heart Beats Rapidly**) (i.e., running, jogging):

_____ times per week without dog **STRENWT**

_____ average minutes each time without dog **STRENWM**

_____ times with dog with dog **STREWTD**

_____ average minutes each time with dog **STREWMD**

**Coding**: 000 to 998 (missing=999) times per week for 000 to 998 minutes (missing=999)

**Moderate Walking (Not Exhausting)** (i.e., fast walking):

_____ times per week without dog **MODWT**

_____ average minutes each time without dog **MODWM**

_____ times with dog with dog **MODWTD**

_____ average minutes each time with dog **MODWMD**

**Coding**: 000 to 998 (missing=999) times per week for 000 to 998 minutes (missing=999)

**Mild Walking (Minimal Effort)** (i.e., easy walking):

_____ times per week without dog **MINWT**

_____ average minutes each time without dog **MINWM**

_____ times with dog with dog **MINWTD**

_____ average minutes each time with dog **MINWMD**

**Coding**: 000 to 998 (missing=999) times per week for 000 to 998 minutes (missing=999)

***International Physical Activity Questionnaire (IPAQ) Craig et al. (2003) [sitting item]***

The next question is about the time you spent **sitting** on weekdays during the **last 7 days**. Include time spent sitting at work, at home, while doing course work and during leisure time. This may include time spent sitting at a desk, visiting friends, reading, or sitting or lying down to watch television.

During the **last 7 days**, how much time did you spend **sitting** on a **week day**?

_____ times per week without dog **SITT**

_____ average minutes each time without dog **SITM**

_____ times with dog with dog **SITTD**

_____ average minutes each time with dog **SITMD**

**Coding**: 000 to 998 (missing=999) times per week for 000 to 998 minutes (missing=999)

**SECTION THREE: EMOTIONAL/MENTAL HEALTH**

***Well-Being Index (WHO-5) (Bonsignore et al. 2001)***

Please indicate how you have been feeling over the last two weeks:

- I have felt cheerful and in good spirits  **WELLBE1**
- I have felt calm and relaxed **WELLBE2**
- I have felt active and vigorous **WELLBE3**
- I woke up feeling fresh and rested **WELLBE4**
- My daily life has been filled with things that interest me **WELLBE5**

**Coding:** at no time (1), some of the time (2), less than half of the time (3), more than half of the time (4), most of the time (5), all of the time (6) (missing=9)

**Depression and Anxiety (PROMIS short form v2.0)**

Please indicate how often you have felt the following in the past seven days:

- I felt fearful **ANXIETY1**
- I found it hard to focus on anything other than my anxiety **ANXIETY2**
- My worries overwhelmed me **ANXIETY3**
- I felt uneasy **ANXIETY4**
- I felt worthless **DEPRESS1**
- I felt helpless  **DEPRESS2**
- I felt depressed **DEPRESS3**
- I felt hopeless  **DEPRESS4**

**Coding**: Never (1), not very often (2), sometimes (3), often (4), most of the time (5) (missing=9)

**Life has Meaning and Purpose *(Developed from interviews)***

- I am hopeful about the future. **MEAN1**
- I set goals for the future. **MEAN2**
- My life has meaning. **MEAN3**
- My life has purpose. **MEAN4**
- I have hope. **MEAN5**

**Coding**: Never (1), not very often (2), sometimes (3), often (4), most of the time (5) (missing=9)

**Life has Structure and Routine (Developed from interviews)**

- My life is fairly structured. **STRUC1**
- My life follows a certain routine. **STRUC2**

**Coding**: Never (1), not very often (2), sometimes (3), often (4), most of the time (5) (missing=9)

**SECTION FOUR: SOCIAL/COMMUNITY TIES**

***Loneliness Scale*** ***(Wood et al., 2005)***

How often in the last 12 months have you:

- Felt lonely? **LONELY1**
- Felt isolated from others? **LONELY2**
- Found it hard to get to know people? **LONELY3**
- Wished that you had more help or support from other people? **LONELY4**

**Coding**: Never (1), not very often (2), sometimes (3), often (4), most of the time (5) (missing=9)

***Networks for Support*** ***(Wood et al. 2005)***

- If you had a serious personal crisis or problem, how many people who live in your neighbourhood (if any) do you feel that you could turn to for comfort and support?

**Coding:** 00-998 as actual numbers (missing=999) **COMMNET**

**Companionship (Short Form) PROMIS v2.0**

- Do you have someone with whom to have fun? **COMPAN1**
- Do you have someone with whom to relax? **COMPAN2**
- Do you have someone with whom you can do something enjoyable? **COMPAN3**
- Can you find companionship when you want it? **COMPAN4**

**Coding**: Never (1), not very often (2), sometimes (3), often (4), most of the time (5) (missing=9)

**Emotional Support (Short Form) PROMIS v2.0**

- I have someone who will listen to me when I need to talk. **EMOTSUP1**
- I have someone to confide in or talk to about myself or my problems. **EMOTSUP2**
- I have someone who makes me feel appreciated. **EMOTSUP3**
- I have someone to talk to when I have a bad day. **EMOTSUP4**

**Coding**: Never (1), not very often (2), sometimes (3), often (4), most of the time (5) (missing=9)

**SECTION FIVE: Dog Ownership**

***Dog Ownership (Wood et al., 2005)***

Do you own a dog? **OWNDOG Coding**: 1=yes, 0=no 9=missing

*If respondents do not own a dog, they will be instructed to go to Section Six on Background Information.*

If yes, do any of your dogs:

have poor physical health? 1=Yes 0=No 9=missing **DOGPOOR**

have mobility issues? 1=Yes 0=No 9=missing **DOGMOBIL**

suffer from chronic pain? 1=Yes 0=No 9=missing **DOGPAIN**

**Sense of Community: Getting to Know People in Neighbourhood through Pets (Wood et al., 2005)**

- Do you talk to other people when walking your dog? **Coding**: 1=yes, 0=no (missing=9) **DOGKNOW1**
- Have you got to know people in your neighbourhood as a result of your pet (for example, through walking your pet or talking to your neighbours about your pet)? **Coding**: 1=yes, 0=no (missing=9) **DOGKNOW2**

**Social Support received by Pet Owners from People met through their Pet(s) (Wood et al., 2005)**

Have you met anyone through your pet who you could:

- talk with about something that was worrying you such as a work or family issue; **Coding**: 1=yes, 0=no (missing=9) **DOGSS1**
- ask for information such as, if they could recommend a tradesperson or restaurant; **Coding**: 1=yes, 0=no (missing=9) **DOGSS2**
- ask for advice; **Coding**: 1=yes, 0=no (missing=9) **DOGSS3**
- ask to borrow something (such as a book or tool), or ask a favor (such as collect mail) or ask for practical help such as getting a ride? **Coding**: 1=yes, 0=no (missing=9) **DOGSS4**

***Dog Attachment (Cutt et al 2008 adapted from Johnson et al.’s Lexington Attachment to Pets Scale (LAPS) “General Attachment” subscale)***

- I talk to my dog. **DOGATT1**
- My dog adds to my happiness. **DOGATT2**
- I talk to others about my dog. **DOGATT3**
- I often play with my dog. **DOGATT4**
- My dog knows how I feel about things. **DOGATT5**
- My dog is considered part of the family. **DOGATT6**

**Coding**: Strongly disagree (1), disagree (2), neither agree or disagree (3), agree (4), strongly agree (5), (missing=9)

***Companionship (Schneider et al. (2010) subscale of Human-Animal Bond (HAB) Scale)***

- If my dog were to die, it would affect me the same as if a close friend died. **DOGCOM1**
- At home, I sit with my dog close to me. **DOGCOM2**
- After a hard day, I like to spend time with my dog. **DOGCOM3**
- I think of my dog as a close friend. **DOGCOM4**

**Coding**: Strongly disagree (1), disagree (2), neither agree or disagree (3), agree (4), strongly agree (5), (missing=9)

**Dog Benefits (Developed for this study from Marcus et al., 2012 qualitative study of benefits of therapy dogs)**

- Spending time with my dog reduces my physical pain. **DOGBEN1**
- Spending time with my dog takes away my stress. **DOGBEN2**
- Petting my dog gets rid of my stress. **DOGBEN3**
- Spending time with my dog is relaxing. **DOGBEN4**
- My dog seems to know when my pain is at its worst. **DOGBEN5**
- My dog provides a positive distraction from my pain. **DOGBEN6**
- My dog takes my mind off my pain. **DOGBEN7**
- My dog is the best treatment for me. **DOGBEN8**

**Coding**: Strongly disagree (1), disagree (2), neither agree or disagree (3), agree (4), strongly agree (5), (missing=9)

**Dog Benefits (developed from Interviews)**

- My dog is not judgemental. **DOGBEN9**
- My dogs listens to me. **DOGBEN10**
- My dog allows me to cry when I need to. **DOGBEN11**
- My dogs knows when I’m in pain. **DOGBEN12**
- My dog provides me with unconditional love. **DOGBEN13**
- My dog asks for nothing in return. **DOGBEN14**
- My dog helps me to get a good night sleep. **DOGBEN15**
- My dog helps me to feel better. **DOGBEN16**

**Coding**: Strongly disagree (1), disagree (2), neither agree or disagree (3), agree (4), strongly agree (5), (missing=9)

***Emotional Benefits*** ***(Schneider et al. (2010) subscale of Human-Animal Bond (HAB) Scale)***

- My dog makes me feel good about myself. **DOGEBEN1**
- My dog helps me see the good things in life. **DOGEBEN2**
- My dog helps me be a better person. **DOGEBEN3**
- Thinking about my dog makes me feel good. **DOGEBEN4**
- My dog helps reduce my anxiety. **DOGEBEN5**
- My dog helps reduce my stress. **DOGEBEN6**
- My dog has improved my mental health. **DOGEBEN7**

**Coding**: Strongly disagree (1), disagree (2), neither agree or disagree (3), agree (4), strongly agree (5), (missing=9)

**Quality of Life for Dog Owners: Stress/Interference Subscale (Oyama et al., 2017)**

Owning a dog:

- Interferes with my ability to go on vacation or leave my house. **DOGQOL1**
- Increases my level of stress. **DOGQOL2**
- Contributes to my pain. **DOGQOL3**
- Interferes with the quality of my sleep. **DOGQOL4**

**Coding**: Never (1), not very often (2), sometimes (3), often (4), most of the time (5) (missing=9)

**Dog Gives Meaning and Purpose (Developed from interviews)**

- My dog makes me hopeful about the future. **DOGMEAN1**
- My dog helps me to reach my goals in life. **DOGMEAN2**
- My dog gives my life meaning. **DOGMEAN3**
- My dog gives my life purpose. **DOGMEAN4**
- My dog gives me a reason to keep going. **DOGMEAN5**

**Coding**: Never (1), not very often (2), sometimes (3), often (4), most of the time (5) (missing=9)

**Dog Provides Structure and Routine (Developed from Interviews)**

- Caring for my dog gives my life structure. **DOGSTRUC1**
- Caring for my dog requires following a certain routine. **DOGSTRUC2**

**Coding**: Never (1), not very often (2), sometimes (3), often (4), most of the time (5) (missing=9)

**SECTION SIX: BACKGROUND INFORMATION**

The next set of questions is intended to collect information on background characteristics of survey participants. This part of the questionnaire is needed to help us understand the characteristics of the people participating in the study. For this reason it is very important information. All information is held in strict confidence and its presentation to the public will be in the form of group data only. If you feel uncomfortable answering a question, you may leave it blank and move onto the next one.

What was your sex at birth? **SEX**

- Male = 1
- Female = 0
- Other (please specify): = 9
- Prefer not to answer = 9 (missing=9)

In what year were you **born**? _______ **BORN**

**Coding**: code actual year (missing = 9999)

**Recoding**: **AGE** = 2017-BORN

What is the highest level of school you have completed? **EDUC**

- Less than high school diploma = 1
- High school graduate = 2
- Some college/university education = 3
- College/university graduate degree = 4
- (missing=9)

How well do you manage on your current available **income**? **INCOME**

**Coding**: ‘impossible or difficult all the time’ = 1, ‘difficult sometimes’ = 2, ‘not too bad’ = 3 and ‘easy’ = 4 (missing=9).

What is your current **housing** situation?

□ Rent =1 □ own = 2 **RENTOWN**

□ house = 1 □ flat/unit/apartment = 2 □ acreage/farm =3

□ other = 4 (missing=9) **HOUSING**

How would you best describe where you currently live? **URBAN**

□ urban = 1 □ large rural centre = 2 □ small rural centre = 3 □ other rural or remote area = 4 (missing=9)

What is your current employment status? (CDC, 2000) **WORK**

- Employed for wages = 1
- Self-employed = 2
- Out of work one year or more = 3
- Out of work less than one year = 4
- Homemaker = 5
- Student = 6
- Retired =7
- Unable to work =8
- (missing=9)

If employed, on average how many **hours per week do you work** in total, including evenings and weekends, at home and at the office? **WKHOURS**

- *1-20 hours per week (1)*
- *21-35 hours per week (2)*
- *36-45 hours per week (3)*
- *46 hours or more per week (4)*
- (NA and missing=9)

**Marital Status**

Which of the following best describes your present situation? (check one only) **MARRIED**

- *Married or co-habiting with someone (including remarried) = 1*
- *Single (Including not married, not cohabitating, widowed, divorced, separated) = 0*
- (missing=9)

If cohabitating/common law, married or remarried: **MARRIYRS**

How long have you been in your current relationship (in years)? _____ *years*

**Coding**: code actual years (missing or NA = 99)

Is your partner employed at this time: *yes (1) no (0)* (missing or NA = 99) **PARTWORK**

Do you have any children currently living at home with you? *Yes (1) No (0)* (missing = 9) **ANYKID**

Do you have any adult dependents currently living at home with you? *Yes (1) No (0)* (missing = 9) **DEPEND**

**Thank you for your time and effort in completing this survey.**

**Your contribution to this study is very much appreciated.**

**If you have any additional comments or thoughts that you would like to add**

**to help us better understand your experiences living with chronic back pain,**

**please use the space provided below.**
