# Supplementary material for: Evaluating the Relationship between Well-Being and Living with a Dog for People with Chronic Low Back Pain: A Feasibility Study
Source: Int J Environ Res Public Health. 2019 Apr 25;16(8):1472. doi: 10.3390/ijerph16081472 (PMC6517939; doi:10.3390/ijerph16081472)
Supplement: Supplementary file 1 [file ijerph-16-01472-s001.zip › ijerph-468433-Supplementary Materials/Supplementary file_Survey.pdf]

SURVEY OF PEOPLE WITH CHRONIC LOW BACK PAIN

The purpose of this survey is to examine the experiences of people with low back pain and whether they differ for people who own a dog compared to those who do not have a dog. The survey consists of six sections asking about: your quality of life, your physical activity, your emotional well-being, your social ties, your dog ownership experiences if you own a dog, and your background characteristics.

Please fill in the responses below that best describe your situation or experiences. If you choose not to answer any question, just leave it blank and move to the next question. You may notice that some of the statements appear very similar to others. This is an established technique used in survey research to more accurately assess peoples' attitudes or experiences by using multiple items. It is important that you answer as many questions as you can, even if they seem quite similar. When you have completed the questionnaire, please place it into the self-addressed, stamped envelope we have provided and place it in the mail. Thank you again for participating in our study and we look forward to your input!

SECTION 1: YOUR QUALITY OF LIFE

This first section asks you about how you have been feeling in terms of your mental and physical health. Please check off the one response that best describes what you are feeling or experiencing.

**Compared to other people your age, how would you rate**	**Poor**	**Fair**	**Good**	**Very Good**	**Excellent**
… your overall health?	○	○	○	○	○
… your physical health?	○	○	○	○	○
… your mental or emotional health?	○	○	○	○	○

In the boxes provided, please fill out how many days during the past 30 days:

- Was your physical health poor (e.g., due to physical illness or injury)? ☐
- Was your mental health poor (e.g., due to stress, depression, or problems with emotions)? ☐
- Did poor physical or mental health keep you from doing your usual activities (e.g., self-care, work, leisure)? ☐

Please circle one number from 0 to 10 that best describes your pain now (or over the last week):

**No Pain**	**0**	**1**	**2**	**3**	**4**	**5**	**6**	**7**	**8**	**9**	**10**	**Worst Possible Pain**

The following questions will help us understand how much your low back pain has affected your ability to manage your everyday activities. Please answer each section by marking one circle that most applies to you. We realize that you may feel that more than one statement may relate to you, but please mark only one circle that best describes your situation.

PAIN INTENSITY

- O The pain comes and goes and is very mild.
- O The pain is mild and does not vary much.
- O The pain comes and goes and is moderate.
- O The pain is moderate and does not vary much.
- O The pain comes and goes and is severe.
- O The pain is severe and does not vary much.

PERSONAL CARE

- O I do not have to change my way of washing or dressing to avoid pain.
- O I do not normally change my way of washing or dressing even though it causes me pain.
- O Washing and dressing increase the pain, but I manage not to change my way of doing it.
- O Washing and dressing increases the pain and I find it necessary to change my way of doing it.
- O Because of the pain I am unable to do some washing and dressing without help.
- O Because of the pain I am unable to do any washing and dressing without help.

LIFTING

YOU MAY SKIP IF YOU HAVE NOT ATTEMPTED LIFTING SINCE THE ONSET OF YOUR LOW BACK PAIN

- O I can lift heavy weights without extra low back pain.
- O I can lift heavy weights but it causes extra pain.
- O Pain prevents me lifting heavy weights off the floor.
- O Pain prevents me lifting heavy weights off the floor, but I can manage if they are conveniently positioned, e.g. on a table.
- O Pain prevents me lifting heavy weights but I can manage light to medium weights if they are conveniently positioned.
- O I can only lift light weights at the most.

WALKING

- O I have no pain walking.
- O I have some pain on walking, but I can still walk normal distances.
- O Pain prevents me from walking long distances.
- O Pain prevents me from walking intermediate distances.
- O Pain prevents me from walking even short distances.
- O Pain prevents me from walking at all.

SITTING

- O Sitting does not cause me any pain.
- O I can sit as long as I need provided I have my choice of sitting surfaces.
- O Pain prevents me from sitting more than one hour.
- O Pain prevents me from sitting more than half an hour.
- O Pain prevents me from sitting more than 10 minutes.
- O Pain prevents me from sitting at all.

STANDING

- O I can stand as long as I want without pain.
- O I have some pain while standing, but it does not increase with time.
- O I cannot stand for longer than one hour without increasing pain.
- O I cannot stand for longer than half an hour without increasing pain.
- O I cannot stand for longer than 10 minutes without increasing pain.
- O I avoid standing because it increases the pain immediately.

Please Continue to Next Page…

SLEEPING

- O I have no pain while in bed.
- O I have pain in bed, but it does not prevent me from sleeping well.
- O Because of pain I sleep only 3/4 of normal time.
- O Because of pain I sleep only 1/2 of normal time.
- O Because of pain I sleep only 1/4 of normal time.
- O Pain prevents me from sleeping at all.

SOCIAL LIFE

- O My social life is normal and gives me no pain.
- O My social life in normal, but increases the degree of pain.
- O Pain prevents me from participating in more energetic activities e.g. sports, dancing.
- O Pain prevents me from going out very often.
- O Pain has restricted my social life to my home.
- O I hardly have any social life because of pain.

TRAVELLING

- O I get no pain while traveling.
- O I get some pain while traveling, but none of my usual forms of travel make it any worse.
- O I get some pain while traveling, but it does not compel me to seek alternative forms of travel.
- O I get extra pain while traveling that requires me to seek alternative forms of travel.
- O Pain restricts all forms of travel.
- O Pain prevents all forms of travel except that done lying down

EMPLOYMENT/HOMEMAKING

- O My normal job/homemaking duties do not cause pain.
- O My normal job/homemaking duties cause me extra pain, but I can still perform all that is required of me.
- O I can perform most of my job/homemaking duties, but pain prevents me from performing more physically stressful activities e.g. lifting, vacuuming, etc.
- O Pain prevents me from doing anything but light duties.
- O Pain prevents me from doing even light duties.
- O Pain prevents me from performing any job or homemaking chore.

Please Continue to Next Page…

SECTION 2: YOUR PHYSICAL ACTIVITY IN A TYPICAL WEEK

*This section asks you about your physical activities. We would like you to think about **a typical week** in answering these questions. Please fill in the blanks or check off the one response that best describes your typical weekly activities.*

How many times on average do you do the following kinds of exercise during your free time? How many minutes do you typically spend each time? Please write your response in the appropriate box.

	Times per week	Minutes each time
**EXAMPLE**	3	20
**Strenuous Exercise (Heart Beats Rapidly)** (i.e., running, jogging, hockey, football, soccer, squash, basketball, cross country skiing, judo, roller skating, vigorous swimming, vigorous long distance cycling).		
**Moderate Exercise (Not Exhausting)** (i.e., fast walking, baseball, tennis, easy cycling, volleyball, badminton, easy swimming, alpine skiing, popular and folk dancing).		
**Mild Exercise (Minimal Effort)** (i.e., yoga, archery, fishing from river bank, bowling, horseshoes, golf, easy walking).		

Considering a typical week, during your leisure time, how often do you engage in any regular activity long enough to work up a sweat (heart beats rapidly)?

O Never O Not very often O Sometimes O Often O A lot of the time

How many times on average do you walk during your free time with or without a dog? How many minutes do you typically spend each time? Please write the appropriate numbers in the rectangles below. If you do not own a dog, please write N/A in the appropriate spaces below.

	Without a dog		With a dog	
	Times per week	Minutes each time	Times per week	Minutes each time
**Strenuous Walking (Heart Beats Rapidly)** (i.e., running, jogging):				
**Moderate Walking (Not Exhausting)** (i.e., fast walking):				
**Mild Walking (Minimal Effort)** (i.e., easy walking):				

How many times in a typical week do you spend sitting with or without your dog? How many minutes do you typically spend each time? Include time spent sitting at work, at home, while doing course work and during leisure time. This may include time spent sitting at a desk, visiting friends, reading, or sitting or lying down to watch television.

Without a dog		With a dog	
Times per week	Minutes each time	Times per week	Minutes each time

Please Continue to Next Page…

SECTION 3: YOUR EMOTIONAL WELL-BEING

*This next section asks you about your emotional well-being. Please indicate which statement is **closest** to how you have been feeling.*

**Over the past 2 weeks…**	**At no time**	**Some of the time**	**Less than half the time**	**More than half the time**	**Most of the time**	**All of the time**
… I have felt cheerful and in good spirits	◯	◯	◯	◯	◯	◯
… I have felt calm and relaxed	◯	◯	◯	◯	◯	◯
… I have felt active and vigorous	◯	◯	◯	◯	◯	◯
… I woke up feeling refreshed and rested	◯	◯	◯	◯	◯	◯
… My daily life has been filled with things that interest me	◯	◯	◯	◯	◯	◯

**Over the past 7 days…**	**Never**	**Not very often**	**Sometimes**	**Often**	**Most of the time**
… I felt fearful	◯	◯	◯	◯	◯
… I found it hard to focus on anything but my anxiety	◯	◯	◯	◯	◯
… My worries overwhelmed me	◯	◯	◯	◯	◯
… I felt uneasy	◯	◯	◯	◯	◯
… I felt worthless	◯	◯	◯	◯	◯
… I felt helpless	◯	◯	◯	◯	◯
… I felt depressed	◯	◯	◯	◯	◯
… I felt hopeless	◯	◯	◯	◯	◯

**In general…**	**Never**	**Not very often**	**Sometimes**	**Often**	**Most of the time**
… I am hopeful about the future	◯	◯	◯	◯	◯
… I set goals for the future	◯	◯	◯	◯	◯
… My life has meaning	◯	◯	◯	◯	◯
… My life has purpose	◯	◯	◯	◯	◯
… I have hope	◯	◯	◯	◯	◯
… My life is fairly structured	◯	◯	◯	◯	◯
… My life follows a certain routine	◯	◯	◯	◯	◯

Please Continue to Next Page…

SECTION 4: YOUR SOCIAL TIES

This section asks about your social and emotional ties and connections with other people.

How often in the past **12 months** have you:	**At no time**	**Some of the time**	**Less than half the time**	**More than half the time**	**Most of the time**	**All of the time**
Felt lonely?	○	○	○	○	○	○
Felt isolated from others?	○	○	○	○	○	○
Found it hard to get to know people?	○	○	○	○	○	○
Wished that you had more help or support from other people?	○	○	○	○	○	○

If you had a serious personal crisis or problem, how many people who live in your neighborhood (if any) do you feel that you could turn to for comfort and support? ☐

**In general…**	**Never**	**Not very often**	**Sometimes**	**Often**	**Most of the time**
I have someone to have fun with.	○	○	○	○	○
I have someone to relax with.	○	○	○	○	○
I have someone to do enjoyable things with.	○	○	○	○	○
I can find companionship when I want it.	○	○	○	○	○
I have someone who will listen to me when I need to talk.	○	○	○	○	○
I have someone to confide in or talk to about myself or my problems.	○	○	○	○	○
I have someone who makes me feel appreciated.	○	○	○	○	○
I have someone to talk to when I have a bad day.	○	○	○	○	○

Please Continue to Next Page…

SECTION 5: YOUR DOG OWNERSHIP EXPERIENCES

This next section asks about your experiences of dog ownership.

Do you own a dog? O **Yes** O **No** ***If you do not own a dog, please turn to Section 6 on page 9.***

If **yes,** do any of your dogs:

… have poor physical health? O **Yes** O **No**

… have mobility issues? O **Yes** O **No**

… suffer from chronic pain? O **Yes** O **No**

Do you talk to other people when out walking your dog? O **Yes** O **No**

Have you got to know people in your neighborhood as a result of your dog (for example, through walking your dog or talking to your neighbors about your dog)? O **Yes** O **No**

Have you met anyone through your dog who you could:

… Talk with about something that was worrying you, such as a work or family issue O **Yes** O **No**

… Ask for information, such as recommending a tradesperson or restaurant O **Yes** O **No**

… Ask for advice O **Yes** O **No**

… Ask to borrow something (such as a book or tool), or ask a favor (such as collect mail), or ask for practical help (such as getting a ride) O **Yes** O **No**

	**Strongly disagree**	**Somewhat disagree**	**Neither agree nor disagree**	**Somewhat agree**	**Strongly agree**
I talk to my dog.	○	○	○	○	○
My dog adds to my happiness.	○	○	○	○	○
I talk to others about my dog.	○	○	○	○	○
I often play with my dog.	○	○	○	○	○
My dog knows how I feel about things.	○	○	○	○	○
My dog is considered part of the family.	○	○	○	○	○
If my dog were to die, it would affect me the same as if a close friend died.	○	○	○	○	○
At home, I sit with my dog close to me.	○	○	○	○	○
After a hard day, I like to spend time with my dog.	○	○	○	○	○
I think of my dog as a close friend.	○	○	○	○	○

	Strongly disagree	Somewhat disagree	Neither agree nor disagree	Somewhat agree	Strongly agree
Spending time with my dog reduces my physical pain.					
Spending time with my dog take away my stress.					
Petting my dog gets rid of my stress.					
Spending time with my dog is relaxing					
My dog seems to know when my pain is at its worst.					
My dog provides a positive distraction from my pain.					
My dog takes my mind off my pain.					
My dog is the best treatment for me.					
My dog is not judgmental.					
My dogs listens to me.					
My dog allows me to cry when I need to.					
My dogs knows when I'm in pain.					
My dog provides me with unconditional love.					
My dog asks for nothing in return.					
My dog helps me to get a good night sleep.					
My dog helps me to feel better.					
My dog makes me feel good about myself.					
My dog helps me see the good things in life.					
My dog helps me be a better person.					
Thinking about my dog makes me feel good.					
My dog helps reduce my anxiety.					
My dog helps reduce my stress.					
My dog has improved my mental health.					
Interfere with my ability to go on vacation or leave my house.					
Increases my level of stress.					
Contributes to my pain.					
Interferes with the quality of my sleep.					
Caring for my dog requires following a certain routine.					

	Never	Not very often	Sometimes	Often	Most of the time
My dog makes me hopeful about the future.					
My dog helps me to reach my goals in life.					
My dog gives my life meaning.					
My dog gives my life purpose.					
My dog gives me a reason to keep going.					
Caring for my dog gives my life structure.					

Please Continue to Next Page…

SECTION 6: YOUR BACKGROUND CHARACTERISTICS

The information in this section is needed to help us understand the characteristics of the people participating in our study and interpret our results. All of your information is confidential and will be presented in aggregate form only.

What was your sex at birth?

- O Male
- O Female
- O Other (please specify): ___________
- O Prefer not to answer

In what year were you born? [ ]

What is the highest level of school you have completed?

- O Less than high school diploma
- O High school graduate
- O Some college/university education
- O College/university graduate degree

How well do you manage on your current available income?

- O Impossible or difficult all the time
- O Difficult sometimes
- O Not too bad
- O Easy

What is your current housing situation?

- O Rent
- O Own
- O House
- O Flat/unit/apartment
- O Acreage/farm
- O Other (please specify): ____________

How would you describe where you currently live?

- O Urban
- O Large rural centre
- O Small rural centre
- O Other rural or remote area

What is your current employment status?

- O Employed for wages
- O Self-employed
- O Out of work one year or more
- O Out of work less than one year
- O Homemaker
- O Retired
- O Student
- O Unable to work

Please Continue to Next Page…

If employed, on average how many hours per week do you work in total, including evenings and weekends, at home and at the office?

- O 1-20 hours per week
- O 21-35 hours per week
- O 46-45 hours per week
- O 46 hours or more per week

Which of the following best describes your present situation?

- O Single (Including not married, not cohabitating, widowed, divorced, separated)
- O Married or co-habiting with someone (including remarried)
    - o If cohabitating/common law, married, or remarried: How long have you been in your current relationship (in years): [ ]

Is your partner employed at this time?

- O Yes
- O No

Do you have any children currently living at home with you?

- O Yes
- O No

Do you have any adult dependents currently living at home with you?

- O Yes
- O No

<u>Thank</u> <u>you</u> for your time and effort in completing this survey.
Your contribution to this study is very much appreciated.

If you have any additional comments that you would like to add to help us better understand your experiences with chronic back pain, please write your comments below or on a separate sheet of paper.
