# Supplementary material for: Evaluating the Relationship between Well-Being and Living with a Dog for People with Chronic Low Back Pain: A Feasibility Study
Source: Int J Environ Res Public Health. 2019 Apr 25;16(8):1472. doi: 10.3390/ijerph16081472 (PMC6517939; doi:10.3390/ijerph16081472)
Supplement: Supplementary file 1 [file ijerph-16-01472-s001.zip › ijerph-468433-Supplementary Materials/Supplementary file_Table 1.docx]

Evaluating the relationship between well-being and living with a dog for people with chronic low back pain: A feasibility study

Eloise C.J. Carr*

*Faculty of Nursing, Professional Faculties Building, 2500 University Drive NW, Calgary, Alberta, Canada T2N 1N4 email:ecarr@ucalgary.ca*

Jean E. Wallace

*Department of Sociology, Faculty of Arts, University of Calgary, 2500 University Drive NW, Calgary, Canada T2N 1N4 email:jwallace@ucalgary.ca*

Rianne Pater

*Faculty of Nursing, Professional Faculties Building, 2500 University Drive NW, Calgary, Alberta, Canada T2N 1N4 email: rpater@ucalgary.ca*

Douglas P. Gross

*Department of Physical Therapy, Rehabilitation Medicine, University of Alberta, 3-28 Corbett Hall, 8205 114St., Edmonton, Alberta, Canada T6G 2G4 email: dgross@ualberta.ca*

* Corresponding author:

Evaluating the relationship between well-being and living with a dog for people with chronic low back pain: A feasibility study

**Background:** Chronic low back pain is a significant societal and personal burden that negatively impacts quality of life. Living with a pet has been associated with health benefits such as greater physical activity, less disability, improved mental health and more social ties to the community.

**Aims:** To evaluate the feasibility of surveying people with chronic low back pain to assess the relationship between living with a pet and well-being.

**Methods:** A mail-out survey was sent to 210 adult patients with chronic low back pain attending a chronic pain program. Measures of quality of life, pain, physical activity, emotional health, social ties and dog ownership were included. Feasibility was assessed by examining the response rate to the survey, and responses to established and newly developed measures. Feasibility was also examined in terms of the potential relationships between dog ownership and a number of key well-being variables for this particular patient population.

**Results:** There were 56 completed surveys returned (n=36 non-dog owners and n=20 dog owners). The established, adapted and newly developed scales revealed promising results. Dog owners reported lower pain, fewer depression and anxiety symptoms, and less loneliness than non-dog owners.

**Conclusions:** Survey burden may have been too high for this population. Living with a dog may have positive effects on well-being for people with chronic pain. The findings from this feasibility study will inform a general population survey, to be conducted with a larger, more representative sample of people living with chronic pain.

Keywords: Chronic pain; well-being; depression, physical activity; dog ownership

# Introduction

Chronic pain is one of the most important current and future causes of morbidity and disability across the world ^1; 2^. In the United States an estimated 20.4% of U.S. adults have chronic pain and 8.0% of U.S. adults have debilitating chronic pain^3^. In Europe, chronic pain of moderate to severe intensity occurs in 19% of adult Europeans ^4^. One in 5 Canadians experience chronic pain, which costs more than cancer, heart disease, and HIV combined ^5^, with costs related to job loss and sick days estimated to be $37 billion per year ^6^. Chronic pain is defined as pain that lasts longer than three months and is associated with significant distress and disability, including reduced physical activities and social role ^7^.

Back and neck pain are the leading global reasons for disability^2^. In the United States, low back pain (LBP) is the most common type of chronic pain and the leading cause of disability in people under 45 years of age^8^ . In Canada, the lifetime prevalence of LBP has been estimated as 84%, with 62% of respondents reporting LBP in the last year ^9^. As already noted, the healthcare and personal costs can be enormous. People living with chronic LBP often report not only living with pain and a loss of physical function, but also a reduced social life, poor psychological well-being ^10^ and poor sleep that is known to reduce pain thresholds ^11^. The medical, social and economic costs of chronic LBP are so large that it has been regarded as an epidemic ^12^. A recent series of articles in the *Lancet* issued a ‘Call for Action’ to change how back pain is cared for ^13^. A key recommendation is to move away from a biomedical model of care towards more “person-centered care focusing on self-management and healthy lifestyles as a means of restoring and maintaining function and optimizing participation.” Novel and cost-effective strategies were recommended; especially those that can be undertaken within community settings and that are easily translated to low- and middle-income countries.

The connection between human and animal health is garnering significant attention in both human and veterinary medicine, with a focus on the concept of the Human-Animal Bond (HAB), and how it relates to healthcare ^14^. Owning a companion dog provides several benefits to owners’ well-being. We use the term well-being in the broadest sense to encompass physical, mental, and social domains of health to encourage a more holistic approach to disease prevention, disease management and health promotion ^15^. Specifically, dog ownership has been associated with: improved physical activity ^16-19^, better mental well-being ^20-22^, greater connectedness to the community or ‘social capital’ ^23; 24^ and reduced medical costs ^25^. The benefits are so positive that dog-walking has been encouraged as a population outreach activity for health promotion and disease prevention ^16^. A companion animal (e.g. cats, dogs) generally includes any animal that shares its life with a human care-giver ^26^. However, we are particularly interested in people who own and live with a dog as they are more researched than other companion animals, more likely to be involved in physical activities outside the home, and are often identified as a member of the family ^27^.

We conducted qualitative interviews with people who had chronic pain and lived with a dog and our findings suggest there were physical, emotional and social benefits that improved their well-being ^28^. We are particularly interested in the potential benefits of owning a dog, where there is a close bond to the dog, and the role this may play in the self-management of chronic LBP. The aim of this study was to evaluate the feasibility of surveying people with chronic LBP to empirically assess the relationship between dog ownership and well-being for people with chronic LBP. Specifically, the primary objectives were to ensure that: (1) the recruitment strategy was effective for this population ^29^; (2) the data collection instrument (paper questionnaire) was appropriate and patient burden was not an issue; and (3) the key variables were valid and reliable for this population. The secondary objective was to explore whether a relationship exists between dog ownership and well-being (physical, mental and social domains) for people with chronic LBP.

# Materials and Methods

***Study design***

We developed a survey instrument from reviews of the relevant literature in combination with the results of qualitative interviews that we carried out with people with chronic pain who own a dog^28^. The survey included measures on quality of life, physical health and functioning, emotional/mental health, social/community ties and dog ownership. The study received ethical approval from the University of Calgary Conjoint Health Research Ethics Board (#REB17-0829).

## Participants

Patients referred to a community multidisciplinary chronic pain program in western Canada were invited to participate if they had current addresses in the regional patient demographics system and met study inclusion criteria. Inclusion criteria were: participants must be ≥18 years, have a diagnosis of chronic LBP present for ≥ 6 months, and a pain score of ≥4 on a Numerical Rating Scale (NRS, 0-10) at the time of invitation to the study. Potential participants were identified from the clinic database using the diagnostic code for low back pain (ICD10 code M54.5).

The average sample size for a feasibility or pilot study has been determined to be 30 participants per group ^30^. Response rates to surveys can be variable but population surveys of chronic pain patients have achieved responses between 52% ^31^ and 62% ^32^. We, therefore, estimated that a sample of 210 patients was adequate for this feasibility study. A data analyst at the pain program wrote a script to randomly identify patients at different stages of their chronic LBP journey including: those on a waitlist to see a physician at the program (n=52); those on a waitlist to see a physician at the program and have participated in group sessions (n=53); those actively being seen by a physician (n=53); and those discharged after completing the program (n=52).

## Survey development

In developing the survey, existing measures of the key variables of interest were first identified from the separate literatures on chronic pain and dog ownership. These measures were chosen for their relevance to the research questions, desirable psychometric properties, and suitability for this target population (see below). Next, we incorporated a number of additional measures that reflected key constructs reported as important from interviews carried out in the first phase of the study^28^. For example, these included how living with a dog provided meaning and purpose to life, routine, companionship, and distraction from chronic pain. Some of these measures already existed in the literature, some were adapted from existing measures, and others were developed for this study based on the participants’ interview responses in stage one of the study. Once we had identified potential measures, they were grouped into four key areas: 1) Well-being and quality of life; 2) physical activity and physical health; 3) emotional and mental health; and 4) social and community ties. In addition, we devoted one section of the survey to a number of specific questions for those owning dogs that was largely influenced by the new concepts identified by the interview phase of the study. Participants were also asked a range of standard demographic questions (e.g. age, sex, family situation, education etc.).

*Well-Being and quality of life*

General health measures and two pain assessment scales were used to assess participants’ well-being and quality of life. Three items from the US National Survey of Families and Household were included that asked participants to rate their overall health, physical health, and mental health, compared to other people their age ^33^. These items used a Likert response scale from 1 (poor) to 5 (excellent) health. These single-item, self-report measures have been shown to be highly correlated with more objective assessments made by physicians and various measures of morbidity ^34^. Second, we included the Healthy Days Measure from the Center for Disease Control and Prevention ^35^. This measure includes three items that ask participants to identify how many days, in the past 30 days, that their physical and mental health was poor, and whether it prevented them from doing their usual activities, self-care, work, or recreation.

Two pain measures were included that assess pain severity and physical functioning with LBP. Pain severity was measured using the pain NRS. The NRS is simple to understand, demonstrates good reliability, and is widely used in clinical and research settings ^36; 37^. The Oswestry Disability Index v2.0 (ODI) is seen as the gold standard for measuring LBP disability ^38^ with excellent reliability and validity ^39^. It is a 10-item scale that measures LBP intensity and the impact of LBP on a range of functions including personal care, lifting, walking, sleeping, and social life, among other functions.

*Physical activity and physical health*

Physical activity was measured using the Godin Leisure Time Exercise Questionnaire (GLTEQ). Participants were asked to recall their average weekly physical activity during their free time over the past month ^40^. Leisure time walking was measured by Brown and Rhodes ^41^ adaptation of the Godin and Shepard ^40^ measure so that it includes time estimates for mild, moderate and strenuous walking with and without a dog.

*Emotional and mental health*

Four measures were included to assess emotional and mental health. Emotional well-being was measured by the WHO-5 Index with five items assessing overall emotional well-being ^42^. The scale has adequate validity both as a screening tool for depression and as an outcome measure in clinical trials, and has been applied successfully across a wide range of study fields ^43^. It has also demonstrated robust psychometric properties and reliability in the evaluation of back pain treatments ^44^. Depression and anxiety were measured by eight items from the Patient-Reported Outcomes Measurement Information System (PROMIS®) short form (v2.0) PROMIS anxiety SF4 and the PROMIS depression SF4 scale ^45; 46^. Two further measures were developed from our interviews with dog owners. One included five items adapted from the PROMIS Parents’ Meaning and Purpose short form (v1.0) scale measuring whether respondents’ life has meaning and purpose. The other included two items developed from the interviews that measures whether respondents’ life has structure and routine.

*Social and community ties*

Social and community ties were measured by four scales. The Loneliness for Support Scale and the Networks for Support Scale were used to measure social and community ties specifically in the dog ownership population ^23; 24; 47^. The Companionship scale and Emotional Support scale (PROMIS, short form, v2.0) each contain 4 items and were used to measure social

functioning ^46; 48^.

*Dog Ownership*

For respondents who owned a dog, we asked additional questions related to the dog’s sex, age, size, primary reason for having the dog, dog health, and attachment. One of the most widely used instruments to measure the attachment pet owners have to their dogs is the Lexington Attachment to Pets Scale (LAPS) ^49; 50^. A series of measures were included to capture a wide array of possible benefits of dog ownership that included support ^23^, emotional benefits (a subscale of the Human-Animal Bond (HAB) Scale ^51^, companionship (a subscale of the Human-Animal Bond (HAB) Scale ^51^, social facilitation, physical benefits, quality of life ^52^, and sense of community ^23^. These measures are based on scales previously used and tested in the literature on companion animals and their connections with and benefits for their owners ^23; 47; 51; 52^. We developed several additional measurs based on the findings from our interviews and another qualitative study on the benefits of therapy dogs ^53^. These items reflected a number of dog benefits such as dog as pain reliever, dog as stress reliever, and dog as unconditional support. The scale ‘dog gives meaning and purpose’ was adapted from the PROMIS (Short form v1.0) Parents’ Meaning and Purpose scale ^46^.

***Patient engagement***

Engaging patients as partners in research can ultimately improve the quality of care and is particularly important for chronic health conditions ^54; 55^. Frameworks such as the Guidance for Reporting Involvement of Patients and Public (GRIPP2) checklist help ensure that reporting patient involvement in research is transparent ^56^. However, the tool has been criticized for assessing the impact of patient engagement in research, rather than the quality of the reporting ^57^. Another tool that is helpful to make the patient involvement in the research process more transparent, is the Patient Engagement in Research Description (PED), that identifies three over-arching categories of who, how, and when ^58^. In this paper we use the PED framework to describe the involvement of patient members in the research process.

The Human Animal Pain Interactions (HAPI) research team has a core team of 15 members, including five patient members and two service dogs. Two members are from Alberta Health Services and the remaining 8 researchers are members of several disciplines including, nursing, sociology, veterinary and human medicine. The patient members all have chronic pain and two live with service dogs. In addition to bringing the patient perspective of living with chronic pain they have specific expertise. For example, three members have been active facilitators for a community patient support group for people with chronic pain who have completed a pain management program. Two patient members have been trained in the Patient and Community Engagement Research (PaCER) method, which is a peer-to-peer inductive research approach designed to create a robust shared patient voice and maximize patient engagement throughout the research process ^59; 60^. The ‘how’ of engaging patient members was achieved by including them as equal members of the research team. They were invited to attend meetings, included in email communications and invited to contribute on specific items that were important to the patient perspective. For example, prior to dissemination, the survey was reviewed by two patient members who lived with service dogs. As subject matter experts, they offered feedback on the questionnaire in terms of the clarity and relevance of measures, as well as the ease and timing of completing the questionnaire.

***Data collection***

A mail-out package was sent to survey participants with a paper copy of the survey, a consent form and a pre-paid return envelope. A recent systematic review demonstrated that paper surveys had significantly higher response rates than electronic surveys and older adults are less likely to be regular internet users ^61^. A review comparing paper and web-based surveys suggested that paper surveys had an average response rate of 56% compared to 33% for online surveys ^62^. Reminder letters were sent as follow up three weeks later. Data collection took place between November 2017 and January 2018.

***Data analysis***

In analyzing the survey data, five types of analyses were carried out. First, descriptive analyses of all of the items were analyzed. Frequencies for all of the items were examined to see whether survey participants are using all of the response options on each item and the range of scores of each item. In addition, the completion rate on each item was noted. Then descriptive information was generated to determine characteristics of the sample in terms of their well-being/quality of life, physical activity and physical health, emotional/mental health, social/community ties, dog ownership experiences and attitudes and demographic variables. Second, scale development and exploratory factor analysis (EFA) were carried out to empirically examine the latent structure among the newly developed measures for the dog ownership part of the questionnaire. This included, for example, measures of the benefits of dog ownership such as dog as pain reliever, dog as stress reliever, and dog as unconditional support. Due to the small sample size for the dog owners who completed the survey (N=20), only items expected to load on a single underlying construct were entered in order to see whether all of the items loaded on a single factor and did not cross load on another factor. For example, all five items expected to measure the dog benefit of “dog a pain reliever” were entered together but they were not entered with the other dog benefits items tapping “dog as stress reliever” or “dog as unconditional support”. Retention of items was based on factor loadings of ≥0.30 where loadings did not cross load on another factor. Items that failed to meet these criteria were then removed from the scale. Scale scores were computed by summing the respondents’ scores for each item and dividing by the number of items. Third, scale confirmation and principal confirmatory factor analysis (CFA) was used to confirm the factor structure of already existing measures, which were completed by all 56 survey participants. For example, the WHO Well-Being Index, and the PROMIS scales were subject to confirmatory factor analysis to determine whether they loaded on a single factor as expected. The same factor loading criteria outlined above were used to interpret the factor structures. Similarly, scale scores represent the mean scores of the relevant items. Fourth, the internal consistency, or reliability, of scale items was examined. To do so, Cronbach’s alphas were estimated for the items included in each scale. Fifth, mean difference tests were carried out by comparing the average scores of dog owners (N=20) with non-dog owners (N=36). In addition, zero-order correlations were also estimated to examine the relationships between pain severity, physical health and functioning, social/community ties and depression.

# Results

The results are organized around the objectives regarding the recruitment strategy, the appropriateness of the data collection instrument, issues related to the measures of key variables, and the relationship between dog ownership and well-being.

***Recruitment and Response Rate***

A total of 56 completed surveys were returned from a mailing out to 210 to people with chronic back pain who had attended a community multidisciplinary pain program in western Canada. Of the 210 surveys mailed out, 7 were returned to sender as the recipient was no longer residing at the address.

The process of recruitment worked well but the 27% response rate was less than anticipated. However, comparing the demographic characteristics between the two groups, dog owners and non-owners, shows that they did not differ significantly by sex, age, education, available income, housing situation, urban/rural location, employment status or marital status (Table 1). Whilst this is a lower than anticipated response rate we suggest that the length of the questionnaire did not overly deter participants from responding, despite having 174 items for dog-owners and 117 items for non-dog owners. We were also surprised to find that many respondents had provided additional written comments. At the end of the survey, respondents were invited to include any additional comments or thoughts that they believed could assist the researchers to better understand the experiences of living with chronic back pain. Of the 56 surveys returned, 16 survey respondents had included substantive feedback, defined as any additional comments or thoughts that were greater than two sentences, totalling 2,842 words. Seven (44%) of the additional comments were made by dog owners while the remaining nine (66%) were made by non-dog owners. Several comments made by non-dog owners related to a time when they previously owned a dog and the positive impact of that relationship. Non-dog owners also spoke about the financial concerns of owning a dog. Comments made by dog-owners also endorsed the positive relationship with their dog but several mentioned the challenges of walking their dog on icy sidewalks in the winter.

***Sample characteristics***

The 56 completed surveys included 36 non-dog owners and 20 dog owners. The participants included 21 men and 35 women. Most (67.3%) were married or in a common law relationship and 19.6% had a child under 18 years of age living at home. They ranged in age from 26 to 82 years (M = 56, SD = 13.08). In terms of employment, 48.2% were unable to work, 19.6% were retired, 17.9% were employed or self-employed and 14.3% were out of work or a homemaker. The majority had some post-secondary education (72.7%). Comparisons were made between the dog owners and non-dog owners on all demographic variables and none were significantly different at the .05 level (results available from authors).

***Completion Rate***

The data collection instrument was a 10-page paper questionnaire. While it appeared appropriate, some participants may have been deterred by its length. In terms of analyzing the completion rates to specific sections, participant fatigue could have been an issue for the measurement of physical activity (34 items) which required too much detail and calculations (missing responses varied between 3 to 43 and only 23% of participants fully completed this section). This measure is popular in dog walking research where one of the primary goals is to assess the link between owning a dog and frequency and intensity of physical activity. For future surveys of people living with chronic pain, we would reduce this burden by using the 4-item short-form version of physical function from the PROMIS survey. It demonstrates good reliability and validity from chronic musculoskeletal pain and has been recommended for clinical research in low back

pain ^63^.

***Validity and Reliability of Measures***

All pre-existing scales were found to represent a single underlying factor as expected. Upon completion of the factor analyses, reliability of the multiple item measures was assessed using Cronbach’s alpha (α). Values of 0.70 or higher indicate an acceptable level of internal consistency among the items ^64^. All of the pre-existing scales had reliabilities of 0.80 or higher (results available from authors). As shown in Table 1, all of the newly developed multiple item measures also indicate good reliability of 0.70 or higher.

**Please insert Table 1**

***Exploration of the relationships between dog ownership and well-being***

Next we turn to assessing the relationship between dog ownership and well-being to assess the feasibility of exploring this relationship further with a larger sample. Since smaller samples require larger differences and associations to achieve statistical significance ^65^, in addition to p-values at the .05 and .01 levels, we also report significant mean differences and correlations at the .10 level. The results in Table 2 show that despite dog owners reporting lower pain severity scores than non-dog owners, both groups report relatively high pain scores. It is interesting to note that the two groups do not differ significantly in their physical functioning or physical health. Again, these scores suggest that our sample suffers from severe disability in performing daily living activities related to their chronic pain. Turning next to the social and community ties measures, we see that the two groups are similar in their degree of loneliness. The dog owners, however, report less loneliness and they have more social ties in terms of offering companionship and emotional support. Lastly, the depression scores differ significantly such that the dog owners reported fewer depression and anxiety symptoms over the last week before the survey.

**Please insert Table 2**

Table 2 also shows how each of the variables correlates with depression for the entire sample. For this we can see that, as expected, greater pain severity is correlated with greater depression. While the two groups do not differ significantly in physical functioning and health, all of the indicators are significantly related to feelings of depression. Also as expected, loneliness is positively related to depression and companionship and emotional support are negatively related.

Based on the pattern of results in this table, we might tentatively submit that the dog owners’ lower depression scores may be partly accounted for the lower severity of their pain, less loneliness and their greater access to companionship and emotional support from their dog. We conclude that further research in this area is feasible and may offer meaningful results to the existing literature on chronic pain and alternative approaches to chronic pain self-management.

**Discussion**

This study evaluated the feasibility of surveying people with chronic low back pain to assess the relationship between living with a pet and well-being. The findings from this feasibility study will inform a larger population survey and some changes in the protocol. We discuss these findings in relation to the recruitment processes, the survey instrument, the development of new and adapted variables, and the findings pertaining to the relationship between dog ownership and well-being.

The response rate of 27% was lower than expected as prior studies using surveys with chronic pain patients have achieved responses between 52% ^31^. The most likely explanation for this lower than anticipated response could be related to the high level of pain and disability in the sample. Characteristics of patients attending chronic pain programs include poverty, financial stress, severe functional disability and past or current medical conditions ^66^. We observed high scores for disability and the fact that questionnaires continued to be returned over a five-month period. Upon receiving the questionnaire, some may have been deterred by the length of the survey (10 pages) and/or some of the more complex questions as noted above (e.g. physical activity). We were also surprised that more people responded who did not have a dog (n=36) compared to those who did (n=20). However, we had not taken into account that in Canada 32% of households own at least one dog ^67^, and will need to adjust the future sample to reflect these statistics and adjust our sample size accordingly. These statistics are similar to those from other western countries. For example, in the UK 30% of households have at least one dog ^68^. In the US at least half the people who live in the community between the ages of 50 and 74 years have a dog or a cat in their household, with 28% owning a dog ^69^. We were unable to locate any studies suggesting people with chronic pain are less or more likely to have a dog.

The preliminary analyses of the multiple-item scales included in this questionnaire yielded favorable results. Despite the small sample size, the established scales conformed to the expected factor structures, demonstrated high internal reliability and were related to depression consistent with the existing literature (see Table 2). These measures appear to be appropriate in assessing the well-being/quality of life, emotional/mental health, and social/community ties for this population. Whilst the original purpose of PROMIS was for use in clinical trials it has been used across many different chronic disease populations. The PROMIS tools can be used for population surveys and across chronic conditions ^70; 71^

The adapted and original measures of the benefits of dog ownership (i.e., dog as pain reliever, stress reliever, and unconditional support) also yielded promising results. Due to the small sample size of this feasibility study, rigorous validation tests could not be performed but should be the next step with a larger sample. Inclusion of these measures in future research could improve our understanding of the benefits of dog ownership for those with chronic pain.

We explored the relationship between dog-owners and non-dog owners and well-being and found differences between pain scores and depression, but no difference on physical function. Depression in people with chronic pain is common with up to 70% of patients experiencing depressive and anxiety disorders, but the underlying mechanisms are poorly understood ^72^. It has been suggested that disability and social isolation, that often accompanies chronic pain, can lead to depression and anxiety, but also it can work in the opposite direction ^73^. It makes sense that pain severity, companionship and emotional support would then be associated with depression. The relationship between dog ownership and depression is not consistent is not consistent in the literature, with some studies finding dog owners have less depression ^74^ and others finding no difference ^75^. These inconsistent findings are likely due to different measures, different populations and a lack of control for confounding variables. However, further exploration is warranted to understand if dog ownership might be a mediating factor for depression for people with chronic pain, and if so then how. Interestingly we found no difference between the two groups for physical functioning, but given the severity of their chronic pain and resultant disability it is not surprising that these people were not particularly active.

**Future research**

Given that approximately 60% of people with back pain do not seek care within the health care system ^9^, future research using our questionnaire battery should likely involve the general population. Limiting the sample to clinical populations, as we did in this feasibility study, omits people who are coping with their pain and able to function independently in the community. It may be that dog ownership and its associated benefits are factors that facilitate this self-management. A general population survey, conducted with representative sampling and computer-assisted telephone interviews or web-based surveys, would enable us to evaluate the role of dog ownership in the self-management of chronic pain.

**Limitations**

To fully understand an individual’s responses to study variables the study design needs to be longitudinal and ideally include measures over a period prior to owning a dog ^76; 77^. In our study design it might be that people who own a dog are healthier than people who choose not to own a pet, a point that has been made by others ^78^. Pet ownership has been associated with more affluent groups but our socio-economic demographic variables were similar for both dog owners and non-dog owners. A further consideration might be a study design allowing participants to be randomly assigned to groups and self-select pet ownership but this would be ethically and logistically challenging.

**Conclusion**

This study evaluated the feasibility of surveying people with chronic low back pain to assess the relationship between living with a pet and well-being. Whilst the survey items appeared to have adequate measurement properties, the response rate and completion rates suggest the survey burden was high, particularly for this population who are very debilitated by their pain. Our preliminary findings suggest that living with a dog may have positive effects on well-being for people with chronic pain. These findings will inform the next stage of this research program that will involve a larger, more representative population survey of people who have chronic low back pain to assess more rigorously the benefits of dog ownership.

**Acknowledgements**

We would like to thank and acknowledge the assistance of Suzanne Basiuk with data collection. We also thank our patient members Robert and Pamela Pyle for taking the time to provide valuable feedback on the survey. Finally, we thank all the participants who took the time to complete the survey.

**Funding**

The study was funded through a Faculty of Nursing, University of Calgary Interdisciplinary Team Development grant (#10016679).

**Declaration of interest Conflict of interest**

None of the authors has any competing interest in relation to this manuscript.

**References**

1. Rice AS, Smith BH, Blyth FM. Pain and the global burden of disease. Pain. 2016; 157(4):791-796.

2. Global Burden of Disease Study Collaborators. Global, regional, and national incidence, prevalence, and years lived with disability for 301 acute and chronic diseases and injuries in 188 countries, 1990-2013: A systematic analysis for the global burden of disease study 2013. Lancet. 2015; 386(9995):743-800.

3. Dahlhamer J, Lucas J, Zelaya C, Nahin R, Mackey S, DeBar L, Kerns R, Von Korff M, Porter L, Helmick C. Prevalence of chronic pain and high-impact chronic pain among adults - united states, 2016. MMWR Morbidity and Mortality Weekly Report. 2018; 67(36):1001-1006.

4. Breivik H, Collett B, Ventafridda V, Cohen R, Gallacher D. Survey of chronic pain in europe: Prevalence, impact on daily life, and treatment. Eur J Pain. 2006; 10(4):287-333.

5. Reitsma M, Tranmer JE, Buchanan DM, VanDenKerkhof EG. The epidemiology of chronic pain in canadian men and women between 1994 and 2007: Longitudinal results of the national population health survey. Pain Research & Management. 2012; 17(3):

166-172.

6. Schopflocher D, Taenzer P, Jovey R. 2011. The prevalence of chronic pain in Canada. Pain Research & Management. 2011; 16(6):445-450.

7. Treede R-D, Rief W, Barke A, Aziz Q, Bennett MI, Benoliel R, Cohen M, Evers S, Finnerup NB, First MB. et al. A classification of Chronic Pain for ICD-11. Pain. 2015; 156(6):1003-1007.

8. Freburger JK, Holmes GM, Agans RP, Jackman AM, Darter JD, Wallace AS, Castel LD, Kalsbeek WD, Carey TS. The rising prevalence of chronic low back pain. Archives of Internal Medicine. 2009; 169(3):251-258.

9. Gross DP, Ferrari R, Russell AS, Battie MC, Schopflocher D, Hu RW, Waddell G, Buchbinder R. A population-based survey of back pain beliefs in canada. Spine. 2006; 31(18):2142-2145.

10. Snelgrove S, Edwards S, Liossi C. A longitudinal study of patients' experiences of chronic low back pain using interpretative phenomenological analysis: Changes and consistencies. Psychol Health. 2013; 28(2):121-138.

11. Tang NK, Lereya ST, Boulton H, Miller MA, Wolke D, Cappuccio FP. Nonpharmacological treatments of insomnia for long-term painful conditions: A systematic review and meta-analysis of patient-reported outcomes in randomized controlled trials. Sleep. 2015; 38(11):1751-1764.

12. Gatchel RJ. The continuing and growing epidemic of chronic low back pain. Healthcare (Basel). 2015; 3(3):838-845.

13. Buchbinder R, van Tulder M, Oberg B, Costa LM, Woolf A, Schoene M, Croft P. Low back pain: A call for action. Lancet. 2018; 391(10137):2384-2388

14. Takashima GK, Day MJ. Setting the one health agenda and the human-companion animal bond. International Journal of Environmental Research and Public Health. 2014; 11(11):11110-11120.

15. Slabaugh SL, Shah M, Zack M, Happe L, Cordier T, Havens E, Davidson E, Miao M, Prewitt T, Jia H. Leveraging health-related quality of life in population health management: The case for healthy days. Popul Health Manag. 2016; 20(1):13-22

16. Christian H, Bauman A, Epping JN, Levine GN, McCormack G, Rhodes RE, Richards E, Rock M, Westgarth C. 2016. Encouraging dog walking for health promotion and disease prevention. American Journal of Lifestyle Medicine. 2016; 12(3):233-243

17. Thorpe RJ, Simonsick EM, Brach JS, Ayonayon H, Satterfield S, Harris TB, Garcia M, Kritchevsky SB, Health AiaBCS. Dog ownership, walking behavior, and maintained mobility in late life. J Am Geriatr Soc. 2006; 54(9):1419-1424.

18. Westgarth C, Christley RM, Christian HE. How might we increase physical activity through dog walking?: A comprehensive review of dog walking correlates. Int J Behav Nutr Phys Act.2014; 11:83.

19. Dall PM, Ellis SLH, Ellis BM, Grant PM, Colyer A, Gee NR, Granat MH, Mills DS. The influence of dog ownership on objective measures of free-living physical activity and sedentary behaviour in community-dwelling older adults: A longitudinal case-controlled study. BMC Public Health. 2017; 17(1):496.

20. McNicholas J, Gilbey A, Rennie A, Ahmedzai S, Dono JA, Ormerod E. Pet ownership and human health: A brief review of evidence and issues. Brit Med J. 2005; 331(7527):1252-1254.

21. Friedmann E, Son H. The human-companion animal bond: How humans benefit. Vet Clin North Am Small Anim Pract. 2009; 39(2):293-326.

22. Ramirez MTG, Hernandez RL. Benefits of dog ownership: Comparative study of equivalent samples. J Vet Behav. 2014; 9(6):311-315.

23. Wood L, Giles-Corti B, Bulsara M. The pet connection: Pets as a conduit for social capital? Social Science & Medicine. 2005; 61(6):1159-1173.

24. Wood L, Martin K, Christian H, Houghton S, Kawachi I, Vallesi S, McCune S. Social capital and pet ownership - a tale of four cities. SSM - Population Health. 2017; 3:442-447.

25. Headey B, Grabka MM. Pets and human health in germany and australia: National longitudinal results. Social Indicators Research.2007; 80(297).

26. Chur-Hansen A, Stern C, Winefield H. Gaps in the evidence about companion animals and human health: Some suggestions for progress. International Journal of Evidence-Based Healthcare. 2010; 8(3):140-146.

27. Irvine L, Cilia L. More-than-human families: Pets, people, and practices in multispecies households. Sociology Compass. 2017; 11(2):e12455-n/a.

28. Carr E, Wallace J, Hellyer PW, Logan L, Onyewuchi C. Exploring the meaning and experience of chronic pain with people who own a dog: A qualitative study. Anthrozoös. 2018; 31(5):551-565.

29. Arain M, Campbell MJ, Cooper CL, Lancaster GA. What is a pilot or feasibility study? A review of current practice and editorial policy. BMC Medical Research Methodology. 2010; 10:67.

30. Billingham SA, Whitehead AL, Julious SA. An audit of sample sizes for pilot and feasibility trials being undertaken in the united kingdom registered in the united kingdom clinical research network database. BMC Medical Research Methodology. 2013; 13:104.

31. Smith BH, Torrance N, Bennett MI, Lee AJ. Health and quality of life associated with chronic pain of predominantly neuropathic origin in the community. The Clinical Journal of Pain. 2007; 23(2):143-149.

32. Toblin RL, Mack KA, Perveen G, Paulozzi LJ. A population-based survey of chronic pain and its treatment with prescription drugs. Pain. 2011; 152(6):1249-1255.

33. Bumpass LL, Sweet JA. 2005. National Survey of Family and Households Codebook. Ann Arbor, MI: Inter-university Consortium for Political and Social Research

34. Barber J, Muller S, Whitehurst T, Hay E. Measuring morbidity: Self-report or health care records? Family Practice. 2009; 27(1):25-30.

35. Moriarty DG, Zack MM, Kobau R. The centers for disease control and prevention's healthy days measures – population tracking of perceived physical and mental health over time. Health and Quality of Life Outcomes. 2003; 1:37-37.

36. Salaffi F, Stancati A, Silvestri CA, Ciapetti A, Grassi W. Minimal clinically important changes in chronic musculoskeletal pain intensity measured on a numerical rating scale. European Journal of Pain. 2004; 8(4):283-291.

37. Dworkin RH, Turk DC, Wyrwich KW, Beaton D, Cleeland CS, Farrar JT, Haythornthwaite JA, Jensen MP, Kerns RD, Ader DN et al. Interpreting the clinical importance of treatment outcomes in chronic pain clinical trials: Immpact recommendations. Journal of Pain. 2008; 9(2):105-121.

38. Fairbank JC, Pynsent PB. The Oswestry Disability Index. Spine. 2000; 25(22):2940-2952

39. Davidson M, Keating JL. A comparison of five low back disability questionnaires: Reliability and responsiveness. Physical Therapy. 2002; 82(1):8-24.

40. Godin G, Shephard RJ. 1985. A simple method to assess exercise behavior in the community. Canadian Journal of Applied Sport Sciences. 1985; 10(3):141-146.

41. Brown SG, Rhodes RE. Relationships among dog ownership and leisure-time walking in western canadian adults. American Journal of Preventive Medicine. 2006; 30(2):131-136.

42. WHO-Five Well-Being Index (WHO-5). 1998. [accessed 2018 26 February]. http://www.who-5.org/

43. Topp CW, Ostergaard SD, Sondergaard S, Bech P. The WHO-5 Well-Being Index: A systematic review of the literature. Psychotherapy and Psychosomatics. 2015; 84(3):167-176.

44. Volinn E, Yang B, He J, Sheng X, Ying J, Volinn W, Zhang J, Zuo Y. West China hospital set of measures in chinese to evaluate back pain treatment. Pain Med. 2010; 11(5):637-647.

45. Kroenke K, Yu Z, Wu J, Kean J, Monahan PO. Operating characteristics of PROMIS four-item depression and anxiety scales in primary care patients with chronic pain. Pain Med. 2014; 15(11):1892-1901.

46. Cella D, Yount S, Rothrock N, Gershon R, Cook K, Reeve B, Ader D, Fries JF, Bruce B, Rose M. The patient-reported outcomes measurement information system (promis): Progress of an nih roadmap cooperative group during its first two years. Medical care. 2007; 45(5 Suppl 1):S3-S11.

47. Wood L, Martin K, Christian H, Nathan A, Lauritsen C, Houghton S, Kawachi I, McCune S. The pet factor--companion animals as a conduit for getting to know people, friendship formation and social support. PLoS One. 2015; 10(4):e0122085.

48. O'Haire ME, Rodriguez KE. Preliminary efficacy of service dogs as a complementary treatment for posttraumatic stress disorder in military members and veterans. J Consult Clin Psychol. 2018; 86(2):179-188.

49. Johnson TP, Garrity TF, Stallones L. Psychometric evaluation of the Lexington Attachment to Pets Scale (LAPS). Anthrozoös. 1992; 5(3):160-175.

50. Ramírez MTG, Quezada Berumen LdC, Hernández RL. Psychometric properties of the Lexington Attachment to Pets Scale: Mexican version (LAPS-M). Anthrozoös. 2014; 27(3):351-359.

51. Schneider TR, Lyons JB, Tetrick MA, Accortt EE. Multidimensional quality of life and human&#x2013;animal bond measures for companion dogs. Journal of Veterinary Behavior: Clinical Applications and Research. 2010; 5(6):287-301.

52. Oyama MA, Citron L, Shults J, Cimino Brown D, Serpell JA, Farrar JT. Measuring quality of life in owners of companion dogs: Development and validation of a dog owner-specific quality of life questionnaire. Anthrozoös. 2017; 30(1):61-75.

53. Marcus DA, Bernstein CD, Constantin JM, Kunkel FA, Breuer P, Hanlon RB. Animal-assisted therapy at an outpatient pain management clinic. Pain Med. 2012; 13(1):45-57.

54. Batalden M, Batalden P, Margolis P, Seid M, Armstrong G, Opipari-Arrigan L, Hartung H. Coproduction of healthcare service. BMJ Quality & Safety. 2016; 25(7):509-517.

55. Wilson MG, Lavis JN, Gauvin FP. Designing integrated approaches to support people with multimorbidity: Key messages from systematic reviews, health system leaders and citizens. Healthcare Policy. 2016; 12(2):91-104.

56. Staniszewska S, Brett J, Simera I, Seers K, Mockford C, Goodlad S, Altman DG, Moher D, Barber R, Denegri S et al. GRIPP2 reporting checklists: Tools to improve reporting of patient and public involvement in research. Res Involv Engagem. 2017; 3:13.

57. Esmail L, Moore E, Rein A. Evaluating patient and stakeholder engagement in research: Moving from theory to practice. J Comp Eff Res. 2015; 4(2):133-145.

58. Hamilton CB, Leese JC, Hoens AM, Li LC. Framework for advancing the reporting of patient engagement in rheumatology research projects. Curr Rheumatol Rep. 2017; 19(7):38.

59. Marlett N, Shklarov S, Marshall D, Santana MJ, Wasylak T. Building new roles and relationships in research: A model of patient engagement research. Quality of Life Research. 2015; 24(5):1057-1067.

60. Shklarov S, Marshal DA, Wayslak T, Marlett N. “Part of the team”: Mapping the outcomes of training patients for new roles in health research and planning. Health Expectations. 2017; 20(6):1428-1436.

61. Remillard ML, Mazor KM, Cutrona SL, Gurwitz JH, Tjia J. Systematic review of the use of online questionnaires of older adults. J Am Geriatr Soc. 2014; 62(4):696-705.

62. Nulty DD. The adequacy of response rates to online and paper surveys: What can be done? Assessment & Evaluation in Higher Education. 2008; 33(3):301-314.

63. Deyo RA, Katrina R, Buckley DI, Michaels L, Kobus A, Eckstrom E, Forro V, Morris C. Performance of a patient reported outcomes measurement information system (promis) short form in older adults with chronic musculoskeletal pain. Pain Med. 2016; 17(2):314-324.

64. Carmines E, Zeller R. 1979. Reliability and Validity Assessment. Thousand Oaks, California [accessed 2018/05/17]. http://methods.sagepub.com/book/reliability-and-validity-assessment.

65. Norman G. Likert scales, levels of measurement and the "laws" of statistics. Advances in Health Sciences Education:Theory & Practice. 2010; 15(5):625-632.

66. May C, Brcic V, Lau B. Characteristics and complexity of chronic pain patients referred to a community-based multidisciplinary chronic pain clinic au - may, c. Canadian Journal of Pain. 2018; 2(1):125-134.

67. Canadian pet market outlook 2014. Rockville, Maryland 20852; [accessed 02/22/2018]. https://www1.agric.gov.ab.ca/$department/deptdocs.nsf/all/sis14914.

68. Murray JK, Gruffydd-Jones TJ, Roberts MA, Browne WJ. Assessing changes in the uk pet cat and dog populations: Numbers and household ownership. Vet Rec. 2015; 177(10):259.

69. Pruchno R, Heid AR, Wilson-Genderson M. Successful aging, social support, and ownership of a companion animal. Anthrozoös. 2018; 31(1):23-39.

70. Craig BM, Reeve BB, Brown PM, Cella D, Hays RD, Lipscomb J, Simon Pickard A, Revicki DA. US valuation of health outcomes measured using the PROMIS-29. Value in Health. 2014; 17(8):846-853.

71. Fenton BW, Palmieri P, Diantonio G, Vongruenigen V. Application of patient-reported outcomes measurement information system to chronic pelvic pain. Journal of Minimally Invasive Gynecology. 2011; 18(2):189-193.

72. Rayner L, Hotopf M, Petkova H, Matcham F, Simpson A, McCracken LM. Depression in patients with chronic pain attending a specialised pain treatment centre: Prevalence and impact on health care costs. Pain. 2016;157(7):1472-1479.

73. de Heer EW, Gerrits MMJG, Beekman ATF, Dekker J, van Marwijk HWJ, de Waal MWM, Spinhoven P, Penninx BWJH, van der Feltz-Cornelis CM.The association of depression and anxiety with pain: A study from nesda. PLoS ONE. 2014; 9(10):e106907.

74. Bao KJ, Schreer G. Pets and happiness: Examining the association between pet ownership and wellbeing. Anthrozoös. 2016; 29(2):283-296.

75. Bradley L, Bennett PC. Companion-animals’ effectiveness in managing chronic pain in adult community members. Anthrozoös. 2015; 28(4):635-647.

76. González Ramírez MT, Landero Hernández R. Benefits of dog ownership: Comparative study of equivalent samples. Journal of Veterinary Behavior. 2014; 9(6):311-315.

77. Wells DL. Domestic dogs and human health: An overview. British Journal of Health Psychology. 2007; 12(1):145-156.

78. Herzog H. The impact of pets on human health and psychological well-being:Fact, fiction, or hypothesis? Current Directions in Psychological Science. 2011; 20(4):236-239.

**Table l. Original and Adapted Measures Developed for Dog Owners (N=20)**

| **Scale**  (Cronbach’s α) | **Items** | **Response Set** | **Item Source** |
| --- | --- | --- | --- |
| **Dog as Pain Reliever** (α=0.72) | Spending time with my dog reduces my physical pain.  My dog seems to know when my pain is at its worst.  My dog provides a positive distraction from my pain.  My dog takes my mind off my pain.  My dog knows when I’m in pain.* | 1=strongly disagree to 5=strongly agree | Adapted from Marcus et al., 2012) themes verbalized by therapy dog participants  (Table 4)  * Original item |
| **Dog as Stress Reliever** (α=0.83) | Spending time with my dog helps bring me away from my stress.  Petting my dog gets rid of my stress.  My dog’s attitude and responses are soothing.* | 1=strongly disagree to 5=strongly agree | * Adapted from Marcus et al., 2012) themes verbalized by therapy dog participants  (Table 4)  * Original item |
| **Dog as Unconditional Support** (α=0.72) | My dog is not judgemental.*  My dogs listens to me.*  My dog allows me to cry when I need to.*  My dog provides me with unconditional love.*  My dog asks for nothing in return. * | 1=strongly disagree to 5=strongly agree | * Original item |
| **Dog Gives Meaning and Purpose** (α=0.95) | My dog makes me hopeful about the future.  My dog helps me to reach my goals in life.  My dog gives my life meaning.  My dog gives my life purpose.  My dog gives me a reason to keep going.* | 1=never to 5=most of the time | Adapted from PROMIS Parent Proxy Bank (2015) V1.0 Meaning and Purpose Short Form 4A  * Original item |
| **Dog Provides Structure and Routine** (α=0.81) | Caring for my dog gives my life structure.*  Caring for my dog requires following a certain routine. * | 1=never to 5=most of the time | * Original item |

* Original items developed from interview [28]

**Table 2. Comparison of Dog Owners (N=20) versus Non-Dog Owners (N=36) and Zero-Order Correlations with Depression**

|  | **Dog Owners**  **(N=20)**  **Mean (SD)** | **Non-Dog Owners**  **(N=36)**  **Mean (SD)** | **Correlation with Depression (N=56)** |
| --- | --- | --- | --- |
| NPI Pain Severity  (Range=0-10) | 6.40 (1.67) | 7.00 (1.45)† | 0.34* |
|  |  |  |  |
| Physical Functioning (Range=0-100)^a^ | 56.95 (11.23) | 56.81 (15.42) | 0.32* |
| - Pain Intensity (Range=0-5) | 3.65 (0.93) | 3.74 (0.95) | 0.26† |
| - Pain Walking (Range=0-5) | 2.65 (0.93) | 2.94 (0.92) | 0.38* |
| Days of Poor Physical Health (Range=0-31) | 19.56 (10.66) | 18.81 (11.72) | 0.29* |
|  |  |  |  |
| Loneliness (Range=1-5) | 2.81 (1.38) | 3.32 (1.51)† | 0.64** |
| Companionship (Range=1-5) | 3.62 (1.15) | 2.99 (1.27)* | -0.49** |
| Emotional Support(Range=1-5) | 3.64 (0.98) | 3.24 (1.26)† | -0.59** |
|  |  |  |  |
| Depression (Range=1-5) | 2.14 (0.79) | 2.73 (1.10)** |  |
|  |  |  |  |

† significant at the .10 level; * significant at the 0.05 level; ** significant at the .10 level

^a^ A score of 41%-60% on the Modified Oswestry Low Back Pain Questionnaire indicates “severe disability” where daily living activities are affected by pain.
