# Supplementary material for: Evaluating the Relationship between Well-Being and Living with a Dog for People with Chronic Low Back Pain: A Feasibility Study
Source: Int J Environ Res Public Health. 2019 Apr 25;16(8):1472. doi: 10.3390/ijerph16081472 (PMC6517939; doi:10.3390/ijerph16081472)
Supplement: Supplementary file 1 [file ijerph-16-01472-s001.zip › ijerph-468433-Supplementary Materials/Supplementary file_Table 2.docx]

Table 2. Comparison of Dog Owners (N=20) versus Non-Dog Owners (N=36) and Zero-Order Correlations with Depression

|  | **Dog Owners**  **(N=20)**  **Mean (SD)** | **Non-Dog Owners**  **(N=36)**  **Mean (SD)** | **Correlation with Depression (N=56)** |
| --- | --- | --- | --- |
| NPI Pain Severity  (Range=0-10) | 6.40 (1.67) | 7.00 (1.45)† | 0.34* |
|  |  |  |  |
| Physical Functioning (Range=0-100)^a^ | 56.95 (11.23) | 56.81 (15.42) | 0.32* |
| - Pain Intensity (Range=0-5) | 3.65 (0.93) | 3.74 (0.95) | 0.26† |
| - Pain Walking (Range=0-5) | 2.65 (0.93) | 2.94 (0.92) | 0.38* |
| Days of Poor Physical Health (Range=0-31) | 19.56 (10.66) | 18.81 (11.72) | 0.29* |
|  |  |  |  |
| Loneliness (Range=1-5) | 2.81 (1.38) | 3.32 (1.51)† | 0.64** |
| Companionship (Range=1-5) | 3.62 (1.15) | 2.99 (1.27)* | -0.49** |
| Emotional Support(Range=1-5) | 3.64 (0.98) | 3.24 (1.26)† | -0.59** |
|  |  |  |  |
| Depression (Range=1-5) | 2.14 (0.79) | 2.73 (1.10)** |  |
|  |  |  |  |

† significant at the .10 level; * significant at the 0.05 level; ** significant at the .10 level

^a^ A score of 41%-60% on the Modified Oswestry Low Back Pain Questionnaire indicates “severe disability” where daily living activities are affected by pain.
